# Quantitative Determination of 7 Saikosaponins in Xiaochaihu Granules Using High-Performance Liquid Chromatography with Charged Aerosol Detection

**DOI:** 10.1155/2021/6616854

**Published:** 2021-02-09

**Authors:** Aoxue Liu, Tongtong Xu, Wenning Yang, Dandan Zhou, Yiwei Sha

**Affiliations:** ^1^R&D Department, GenChim Testing (Shanghai) Co. Ltd., Shanghai 200131, China; ^2^School of Chinese Materia Medica, Beijing University of Chinese Medicine, Beijing 102488, China

## Abstract

Bupleuri Radix (Chaihu, in Chinese) is the principal drug in Xiaochaihu granules (XGs) that is a famous Chinese medicine preparation in China. Since previous analytical methods have not focused on the multiactive saikosaponins of Chaihu, it is difficult to effectively control the quality of XG on the market. In this manuscript, the simultaneous determination of 7 saikosaponins (saikosaponins C, I, H, A, B_2_, G, and B_1_) in XG by HPLC with charged aerosol detection (CAD) and confirmation by LC-Q-Orbitrap HRMS were described. The saikosaponins were purified on an SPE cartridge and determined on a Waters CORTECTS C18 column (4.6 mm × 150 mm, 2.7 *μ*m) by gradient elution using 0.01% acetic acid aqueous solution and acetonitrile. The results showed good linearity with the *r*^2^ values higher than 0.998 for all analytes. The average recoveries at three different concentration levels ranged from 80% to 109% and the intraday and interday precision (relative standard deviations, RSD%) were in the range of 1.0%∼1.9% and 1.4%∼2.1%, respectively. The established HPLC-CAD method was subsequently applied to 15 batches of XG to investigate the batch-to-batch consistency and controllability. The proposed method could potentially be used for the quality control of XG and also be helpful in the quality evaluation of Chaihu and its related preparations.

## 1. Introduction

Xiaochaihu granules (XGs) are derived from a traditional Chinese medicine (TCM) classical formula Xiaochaihu-tang (named Sho-saiko-to in Japanese). It is a modern Chinese medicine preparation composed of 7 herbs including Bupleuri Radix (Chaihu, in Chinese), Scutellaria radix (Huangqin), Codonopsis radix (Dangshen), Glycyrrhiza radix (Ganchao), Pinellia tuber (Banxia), Jujube fruit (Dazhao), and Ginger rhizome (Shengjiang). It has the function of inducing sweat to dispel heat, soothing liver, and harmonizing stomach [1]. Nowadays, XG produced by dozens of manufactures has been sold as an over-the-counter medicine and widely used for the treatment of influenza, bronchitis, pneumonitis, hepatic fibrosis, and gastrointestinal diseases in clinical practice [2, 3]. Due to its good efficacy, few side effects, and convenient administration, XG has been one of the best-selling anticold medicines and used to treat roughly millions of patients annually in China.

Chaihu is the principal drug in XG that plays a major therapeutic role in the treatment of the main disease and syndrome. Literature studies reported that saikosaponins have many pharmacology activities, such as antiviral, antibacterial, antihepatitis, and immunomodulating effects [4–6], and the major active constituents are saikosaponin A (SSa), saikosaponin C (SSc), and saikosaponin D (SSd) in Chaihu [7, 8]. Since the unstable allyl oxide bonds could be broken under mildly acidic condition or by heating, they might be partially or completely converted into saikosaponin B_1_ (SSa ⟶ SSb_1_), saikosaponin I or H (SSc ⟶ SSi or SSh), and saikosaponin B_2_ (SSd ⟶ SSb_2_) accordingly. Because of the lacking of UV chromophores, HPLC-UV [9] is imperfect for saikosaponins analysis. The evaporative light scattering detector (ELSD) [10] and charged aerosol detector (CAD) [10] are usually recommended to analyze those compounds with weak or no UV absorption. Eom [10] compared CAD and ELSD methods in the simultaneous analysis of 10 saikosaponins in Chaihu. However, both methods were shown to be not suitable for saikosaponins analysis in XG due to the low content and multicomponent mutual interference. In addition, mass spectrometry (MS) technology [8, 11] has been mainly focused on the in vivo metabolism study. Bao et al. [8] established an LC-MS/MS method to determine several saikosaponin derivatives present in Chaihu and its phytopharmaceuticals of Xiaochaihu-tang, which could be applied to the DMPK evaluation and study. LC-MS technique has some limitations for large-scale routine analysis of herbal medicine as a result of its expensive price, high maintenance cost, and low penetration. Furthermore, most methods aimed at Chaihu were very difficult to be transferred for XG analysis because of the structural transformation between active components [7, 8]. The sample processing procedure is also a key factor in the analysis. Several novel extraction methods, such as microwave assisted extraction [12], ultrasound assisted extraction [12], ultrasound- and slat-assisted liquid-liquid extraction [13, 14], matrix solid-phase dispersion [15, 16], and vortex-assisted liquid-liquid microextraction [17], have been developed for natural product analysis. Considering the practicability and operability of the quantitative method used for QC laboratories, routine methods including liquid-liquid extraction and solid-phase extraction were compared and evaluated in this paper. The current Chinese Pharmacopeia (ChP) method [1] ignores the assay of saikosaponins and the Japanese Pharmacopoeia (JP) method [18] only focuses on single ingredient SSb_2_. It is widely acknowledged that the synergistic effects of multiple-active components are responsible for the therapeutic effects of TCM preparation. Therefore, the reported methods and current standards are insufficient to effectively ensure the quality of XG.

In this work, 7 saikosaponins (saikosaponins C, I, H, A, B_2_, G, and B_1_) present in XG were unambiguously identified by comparing with reference standards using LC-Q-Orbitrap HRMS. All analytes were purified and concentrated on a C18 SPE cartridge. The established HPLC-CAD method was proved to be specific, reliable, and practical for the quantitative determination of 7 saikosaponins and successfully applied to the quality evaluation of 15 batched of XG thereof.

## 2. Materials and Methods

### 2.1. Chemicals and Reagents

**Figure 1:**
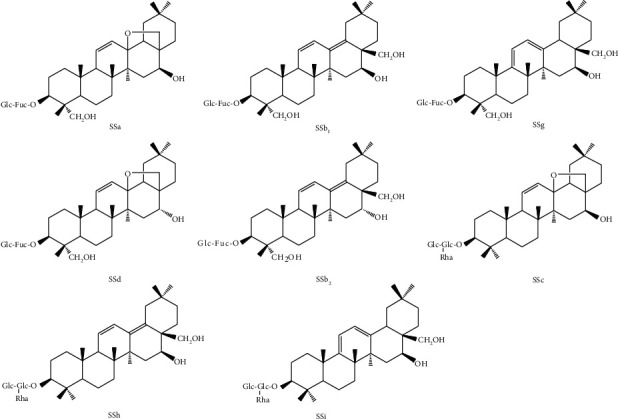
Structures of eight saikosaponins (Glc: *β*-D-glucose; Fuc: L-fucose; Rha: *α*-L-rhamnose).

Saikosaponin A (SSa) and saikosaponin D (SSd) were purchased from the National Institutes for Food and Drug Control (Beijing, China). Saikosaponins C (SSc), I (SSi), H (SSh), G (SSg), B1 (SSb_1_), and B2 (SSb_2_) were purchased from Chengdu Desite Biological Technologies Co., Ltd. (Chengdu, China). Their structures are shown in Figure 1. 15 batches of XG were supplied by Guangzhou Baiyunshan Guanghua Pharmaceutical Co., Ltd. (Guangzhou, China). Sep-Pak C18 SPE cartridges were obtained from Waters (Milford, MA, USA). LC-MS grade acetonitrile and acetic acid were purchased from Fisher (Loughborough, UK). Other reagents were of analytical grade and purchased from Titan Scientific Co., Ltd. (Shanghai, China). Deionized water was prepared by Milli-Q system (Bedford, MA, USA).

### 2.2. Sample Preparation

#### 2.2.1. Reference Standard Solutions

A mix stock standard solution of SSc (0.1 mg·mL^−1^), SSi (0.1 mg·mL^−1^), SSh (0.1 mg·mL^−1^), SSa (0.3 mg·mL^−1^), SSb_2_ (1 mg·mL^−1^), SSg (0.2 mg·mL^−1^), and SSb_1_ (0.5 mg·mL^−1^) was prepared with methanol. A series of mixed standard solutions were obtained by appropriate dilution of the stock solution for plotting calibration curve. All solutions were stored in the refrigerator at 4°C until analysis.

#### 2.2.2. Sample Solution Preparation

An aliquot of 3 g XG dissolved in 6 mL water was loaded onto a C18 SPE cartridge that was preconditioned with 6 mL methanol and 10 mL water sequentially. The cartridge was washed with 10 mL 10% methanol aqueous solution containing 5% concentrated ammonia and followed by 30% aqueous methanol solution. After that, the cartridge was eluted using 10 mL methanol to desorb the analytes. The eluate was evaporated to dryness using a vacuum concentrator. The residue was dissolved with methanol and then transferred into a 2 mL volumetric flask which was brought up to its volume with methanol. All solutions were filtered through a 0.22-*μ*m filter membrane before LC analysis.

### 2.3. LC-MS Condition

The qualitative identification of saikosaponins in XG was carried out using a Q Exactive™ hybrid quadrupole-orbitrap mass spectrometer equipped with a Dionex Ultimate 3000 LC system (Thermo Fisher Scientific, USA). Analytes were detected at 30°C on a Waters CORTECTS C18 column (4.6 mm × 150 mm, 2.7 *μ*m). The mobile phase was comprised of 0.01% acetic acid (A) and acetonitrile (B) with gradient elution program: 0∼9 min, 5–25% B, 9∼16 min, 25–35% B, 16∼23 min, 35–60% B, and 23∼30 min, 60–80% B. The flow rate was 0.3 mL·min^−1^. The heated ESI (H-ESI) source was operated, and MS parameters were optimized as follows: spray voltage, 3.5 kV; sheath gas flow, 40 L/min; aux gas flow, 10 L/min; capillary temperature, 320°C; aux gas heater temperature, 350°C. A full MS/dd-MS^2^ (data dependent MS^2^) method was used for acquisition. The full scan range was from 100 to 1500 in negative ion mode at a resolution of 70000 and 17,500 for MS^2^ scan. MS/MS spectra were fragmented by high-energy collision-induced dissociation (HCD) of normalized collision energy (NCE) values at the levels of 10%, 20%, and 30%. Date acquisition and processing were accomplished with Xcalibur software (version 4.2, Thermo Fisher Scientific, USA).

### 2.4. HPLC-CAD Chromatographic Condition

A Dionex Ultimate 3000 LC system (Thermo Fisher Scientific, USA) consisting of vacuum degasser, quaternary pump, autosampler, thermostatted column compartment, and CAD, connected with Chromeleon software, was employed for quantitative analysis. The column, mobile phase, and column temperature were the same as that of LC-MS condition. The gradient elution program was as follows: 0∼55 min, 30–32% B, 50∼58 min, 32–90% B, and 58∼60 min, 90–30% B, and finally, reconditioning the column with 30% B isocratic for 10 min. The flow rate was 0.8 mL·min^−1^, and the injection volume was 10 *μ*L. The nitrogen inlet pressure for CAD was set to 50 psi, and nebulizer chamber temperature was 50°C.

### 2.5. Validation of the Method

The quantitative HPLC-CAD method was fully validated in terms of specificity, linearity, limit of detection (LOD), limit of quantitation (LOQ), intra- and interday precision, repeatability, and accuracy in accordance with ICH guideline Q2(R1) [19]. Specificity was assessed by comparing the chromatograms obtained from blank solution, mix standard solution, test sample, and test sample without Chaihu. A series of mix standard solutions at six different concentration levels were prepared and plotted the calibration curve, where *y* and *x* represent the logarithm of peak area and logarithm of concentration (*μ*g·mL^−1^). LOD and LOQ were determined by diluting the reference standard solution with methanol when the signal-to-noise rations (S/N) were about 3 : 1 and 10 : 1, respectively. For intra- and interday precision, the mix standard solution was determined in the same day (*n* = 6) and in three consecutive days, respectively. Six replicates as per the test method representing a single batch were prepared and analyzed to demonstrate the repeatability. The precision and repeatability were evaluated by calculating the relative standard deviations (RSDs) of each peak area. The recovery was used to express the accuracy of the method, which was performed by spiking known amounts of the sample to three concentration levels (50%, 100%, and 150%) of the mix standard solution.

## 3. Results and Discussion

### 3.1. Method Optimization

ELSD and CAD are both universal detectors and have obvious advantages in detecting those compounds with weak or no UV absorption. The performance comparison between these two detectors was studied by some researchers [10, 20]. It was demonstrated that CAD was superior in linearity, sensitivity, reproducibility, and peak sharpness. As a result, CAD is used as a valuable alternative to ELSD for pharmaceutical analysis [21–23].

XG is a mixture of multiple herbs and thus has a wide range of complex and diverse chemical components [24–27]. The determination of saikosaponins is difficult because of the low content and multicomponent mutual interference in XG. The sample processing procedure that is able to purify and concentrate the analytes could solve the problem. The liquid-liquid extraction (LLE) using water-saturated n-butanol solution and C18 SPE cartridge were investigated comparatively. It was shown that LLE was unable to remove the major interfering component baicalin present in Huangqin and other impurities thoroughly, while SPE cartridge had obvious superiority in terms of extraction speed, precision, and reliability and was therefore employed for sample preparation. Three brands of SPE cartridges (Waters, Sepax technologies, and Welchrom) were studied, and it was found that Waters cartridge had high cleanup efficiency and good recovery compared with the others. Saikosaponins were fully absorbed on the C18 cartridge when using low proportional methanol in an alkaline solution as the eluent that could get rid of most interfering components. Nevertheless, they were easily desorbed by eluting with a high proportional methanol solution.

The HPLC-CAD chromatographic condition described in the reference literature [10] was attempted initially, but found to be unsuitable for saikosaponins analysis in XG in the aspect of drifting retention times, poor peak shape, low resolution between analytes, and their adjacent peaks. Several gradient programs by using different mobile phase additives (formic acid, acetic acid, and ammonium acetate) were investigated to get a better separation and higher response.

### 3.2. Qualitative Identification of Saikosaponins

**Figure 2:**
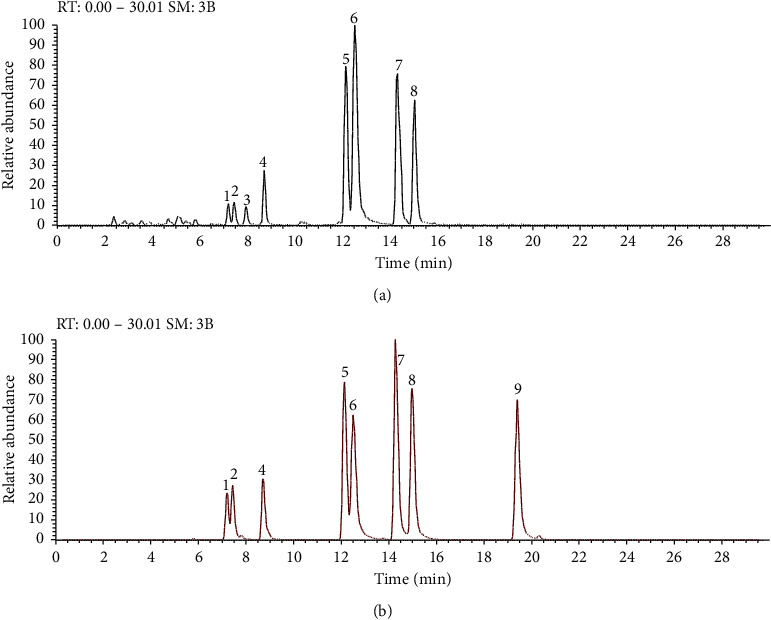
Extracted ion chromatogram (EIC) of the test sample (a) and mixed standard solution (b). (1) SSc, (2) SSi, (3) rotundioside D, (4) SSh, (5) SSa, (6) SSb_2_, (7) SSg, (8) SSb_1_, and (9) SSd.

7 saikosaponins peaks in XG were unambiguously identified by comparing their retention time and UV and MS spectra with those of reference standard solutions. The extracted ion chromatogram (EIC) and MS/MS data are present in Figure 2 and Table 1. The peak 3 showed in Figure 2 was tentatively identified as rotundioside D according to the literature [26]. SSd (peak 9) was not detected in the test sample, probably because it had been entirely transformed into SSb_2_ (peak 6) during the production of XG. This result was in accordance with literature reports [8].

### 3.3. Method Validation

**Figure 3:**
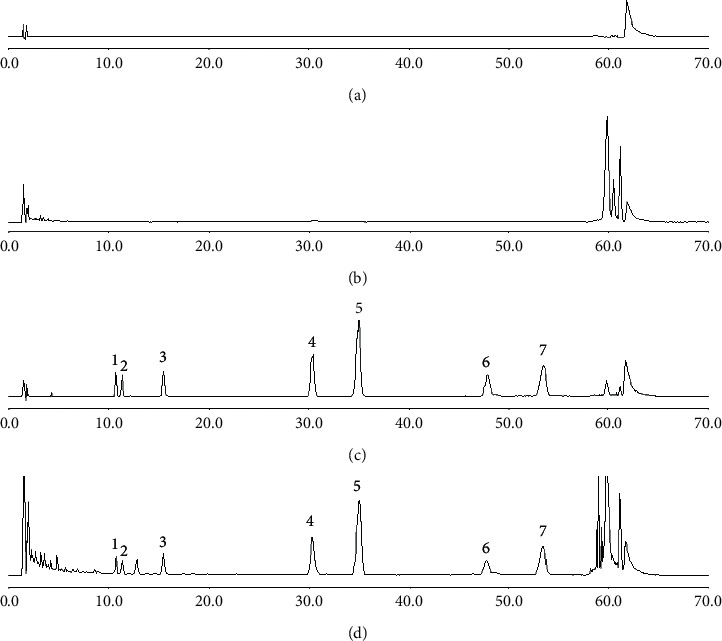
HPLC-CAD chromatograms of seven saikosaponins in XG: blank sample (a), test sample without Bupleuri Radix (b), mixed standard solution (c), and test sample (d). (1) SSc, (2) SSi, (3) SSh, (4) SSa, (5) SSb_2_, (6) SSg, and (7) SSb_1_.

As shown in Figure 3, no interfering peaks were observed at the retention time of target analytes in the test sample, which indicated the method is specific. The linear regression, correlation coefficient (*r*^*2*^), linearity range, LOD, LOQ, intra- and interday precision, repeatability, and recovery of the 7 analytes are listed in Tables 2 and 3. The results showed good linear relationships and high precision. The variations (RSD%) of repeatability were NMT 6.0% for SSc, SSa, SSb_2_, SSg, and SSb_1_ and NMT 8.0% for SSi and SSh. The average recoveries at three different concentration levels were between 80% and 109%. These obtained values illustrated great repeatability and accuracy.

### 3.4. Sample Analysis

**Figure 4:**
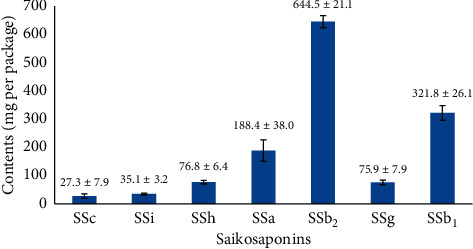
The content of 7 saikosaponins in 15 batches of XG (mg/package, $\bar{x}\pm s$).

The validated HPLC-CAD quantitative method was subsequently applied to determine 7 saikosaponins (SSc, SSi, SSh, SSa, SSb_2_, SSg, and SSb_1_) in 15 batches of XG. The results (mg/package, $\bar{x}\pm s$) are present in Figure 4. The content of saikosaponins in XG was in the order of SSb_2_ > SSb_1_ > SSa > SSh ≥ SSg > SSi > SSc. The high standard deviation values of SSa, SSb_2_, and SSb_1_ among 15 batches probably resulted from the quality difference of Chaihu. Given the structural transformation during processing, the total content of the target analytes in each batch was calculated for quality evaluation. The average content was 1.37 mg per package with a relative standard deviation (RSD%) of 4.4%, which demonstrated acceptable batch-to-batch consistency and controllability. Bao et al. [8] reported that the major saikosaponins contained in the test Xiaochaihu-tang and Sho-saiko-to samples were SSb_2_ (26.9%), SSa (25.8%), SSb_1_ (22.4%), SSg (14.3%), SSc (6.9%), SSh (5.4%), and SSi (2.9%). The difference between our results and literature data was mainly because of different herb extraction processes of XG and Xiaochaihu-tang (Sho-saiko-to).

## 4. Conclusions

In summary, 7 saikosaponins (SSc, SSi, SSh, SSa, SSb_2_, SSg, and SSb_1_) present in XG were unambiguously identified using LC-Q-Orbitrap HRMS and thus selected as quality control components. A specific, sensitive, and accurate HPLC-CAD method in determining the analytes was described in this manuscript. A C_18_ SPE cartridge was used to purify and concentrate the analytes by eluting with a 10% methanol aqueous solution containing 5% concentrated ammonia and a 30% methanol aqueous solution, and then by methanol. The peaks of 7 saikosaponins were well-separated, although most of the analytes are isomeric compounds. This quantitative method could potentially meet the requirement for quality analysis for QC laboratories. Also, the proposed method could to be used for quality evaluation of Chaihu and its related preparations.

## Figures and Tables

**Table 1:** LC-MS/MS data of investigated saikosaponins in XG and mixed standard solution.

No.	Compounds	RT (min)	MS (m/z) [M + CH_3_COO]^−^	Error (ppm)	Formula	MS/MS
1^a^	Saikosaponin C	7.20	985.5381	1.689	C_48_H_78_O_17_	925.5186
2^a^	Saikosaponin I	7.45	985.5379	1.429	C_48_H_78_O_17_	925.5197, 779.4550
3	Rotundioside D	7.96	987.5378	1.728	C_48_H_80_O_17_	927.5322
4^a^	Saikosaponin H	8.72	985.5385	1.094	C_48_H_78_O_17_	925.5201, 779.4578
5^a^	Saikosaponin A	12.14	839.4802	1.362	C_42_H_68_O_13_	779.4572, 617.4080
6^a^	Saikosaponin B_2_	12.52	839.4802	1.593	C_42_H_68_O_13_	779.4599, 617.4064
7^a^	Saikosaponin G	14.31	839.4803	1.593	C_42_H_68_O_13_	779.4609, 617.4037
8^a^	Saikosaponin B_1_	15.04	839.4802	1.362	C_42_H_68_O_13_	779.4663, 617.4097
9^a^	Saikosaponin D	19.41	839.4797	0.887	C_42_H_68_O_13_	779.4602, 617.4061

*Note*. ^a^Unambiguously identified by comparing with reference standards.

**Table 2:** Linearity, LOD, and LOQ data of 7 saikosaponins.

Analytes	Linear regression	*r* ^*2*^	Linear range (*μ*g·mL^−1^)	LOD (*μ*g·mL^−1^)	LOQ (*μ*g·mL^−1^)
SSc	*y* = 0.974*x* + 0.5906	0.9987	2.13–21.27	0.7	1.5
SSi	*y* = 1.0084*x* + 0.5974	0.9993	2.01–20.12	0.5	1.0
SSh	*y* = 1.0178*x* + 0.6200	0.9992	3.16–31.58	0.5	1.0
SSa	*y* = 1.0062*x* +0.6638	0.9990	6.89–68.94	0.3	0.6
SSb_2_	*y* = 0.9089*x* + 0.5575	0.9982	20.71–207.07	0.3	0.6
SSg	*y* = 1.0284*x* + 0.7148	0.9991	5.26–52.64	1.0	2.0
SSb_1_	*y* = 1.0143*x* + 0.5554	0.9995	10.42–104.24	0.3	0.6

**Table 3:** Intraday and interday precision, repeatability, and recovery data of 7 saikosaponins.

Analytes	Precision (RSD%, *n* = 6)		Repeatability (RSD%, *n* = 6)	Recovery (%)	
	Intraday	Interday		Spiked levels (%)	$\bar{X}$ (*n* = 3)
SSc	1.9	1.9	5.2	50	101.1
				100	108.9
				150	92.6

SSi	1.0	1.7	6.0	50	107.5
				100	101.3
				150	93.5

SSh	1.8	1.7	6.8	50	105.1
				100	96.5
				150	90.1

SSa	1.7	2.1	3.6	50	108.7
				100	106.2
				150	92.1

SSb_2_	1.2	1.4	4.4	50	103.9
				100	92.7
				150	82.3

SSg	1.7	1.7	4.4	50	100.5
				100	88.1
				150	79.7

SSb_1_	1.0	2.1	5.3	50	108.7
				100	92.2
				150	84.8

## Data Availability

The data used to support this study were obtained from GenChim Testing (Shanghai) Co., Ltd, Shanghai, China, and are available from the corresponding author upon request.

## Abbreviations

HPLC-CAD: High-performance liquid chromatography with charged aerosol detection
LC-Q-Orbitrap HRMS: Liquid chromatography with hybrid quadrupole-orbitrap mass spectrometer
XG: Xiaochaihu granule
TCM: Traditional Chinese medicine
NMT: Not more than
$\bar{x}\pm s$: Mean ± standard deviation.
